# CAR-T Cell Therapy: From the Bench to the Bedside

**DOI:** 10.3390/cancers9110150

**Published:** 2017-10-31

**Authors:** Vita Golubovskaya

**Affiliations:** Promab Biotechnologies, 2600 Hilltop Drive, Richmond, CA 94806, USA; vita.gol@promab.com; Tel.: +1-510-974-0697; Fax: +1-510-740-3625

CAR (Chimeric Antigen receptor)-T cell therapy is a novel type of therapy that uses engineered T cells with an antibody single-chain variable fragment (ScFv) extracellular domain that binds tumor-associated antigens. CAR-T cells activated with C-terminal to ScFv co-activation domains (CD28, 4-1BB, CD27, and others) and activation domains, inducing T-cell-killing machinery with cytokine and granzyme secretion [1,2]. This causes the killing or apoptosis of target tumor cells [3]. There have been many clinical trials, recently, on hematological cancers that have resulted in successful treatment of leukemia and lymphoma patients [4,5], culminating in FDA approval of Kymriah and Yescarta CAR-T cell therapy products. In this issue, “*CAR-T Cell Therapy against Different Types of Cancer*”, novel findings in CAR-T cell studies and clinical trials against different types of tumors are highlighted and discussed.

The review by Sidra Anwar et al. discusses the importance of quality of life (QOL) outcome analysis, which has an important role in both clinical trials and regulatory drug approvals, especially when survivals are not significantly different compared to standard of care outcomes [6,7]. The authors analyze QOL and social support factors as a tool for evaluating patient fitness and risk, and for serious adverse events (SAE) [8]. Identifying patients with an increased risk for SAEs will improve clinical benefit in clinical trials. The authors analyzed only 92 patients from across 22 clinical trials, but this review provides an interesting approach. The authors suggest that analysis of more patients will allow them to develop predictive decision algorithms to decrease SAE and improve clinical benefits. This approach can be applied to any oncological therapy, including novel CAR-T cellular therapy.

The review by Klampatsa et al. describes clinical studies with CAR-T cells in mesothelioma [9]. Malignant pleural mesothelioma is an aggressive type of cancer starting in mesothelial cells of the pleural cavity [9]. One of the main causes of malignant pleural mesothelioma is prior exposure to asbestos, with an annual incidence of the disease in the USA of about 3300 cases [9]. The authors review potential tumor-associated targets for clinical trials for mesothelioma, such as mesothelin, fibroblast-associated protein (FAP), Pan Erb “T4” (CAR targeting EGFR, Her-2, Her-3 and Her4), 5T4 (oncofetal cell surface glycoprotein), and cell surface proteoglycan chondroitin sulfate proteoglycan 4 (CSPG4) [9]. The approach, which simultaneously targets four tumor-associated antigens with Pan Erb “T4”, is very interesting, and can be expanded to other similar targets in the future.

The review by Sridhar and Petrocca reviews a useful approach to regional delivery of CAR-T cells for treatment of solid tumors [10]. The authors discuss regional delivery of IL13Ralpha 2-CAR-T in glioblastoma and CEA-CAR-T in hepatic colorectal metastases or peritoneal carcitomatosis, as well as Erb-CAR-T in head and neck carcinoma, and mesothtelioma [10]. This type of approach increases the safety of CAR-T cell therapy, and shows high efficacy in clinical trials. This is a highly promising approach for solid cancers, and can be expanded to other targets, and cancers.

Another original study from Hombach and Abken (Cologne, Germany) shows that CD4+CD25− but not CD4+CD25+ (T regulatory, Treg) were able to kill cancer cells [11]. The low cytotoxic activity of CAR-Treg allows use of these cells to inhibit autoimmune responses and treat chronic autoimmune diseases [11]. CAR-Treg cells can also be used for allogenic CAR-T cell generation without the risk of inducing graft-versus-host response.

Finally, the report from Golubovskaya et al. demonstrates a new CD47-CAR-T efficacy against ovarian, pancreatic, and hepatocellular carcinoma, as well as lung cancer and melanoma [12]. The authors used mouse and humanized CD47-CAR-T cells, which effectively killed CD47+ cancer cells but not CD47− cells. The efficacy of CD47-CAR-T cells was significantly higher than Mock CAR-T cells in a pancreatic BxPC3 xenograft NSG mice model [12].

In summary, this issue demonstrates original and novel approaches of CAR-T cells against different types of tumors, e.g., glioblastoma, head and neck, colorectal hepatic metastases, pancreatic, and mesothelioma; demonstrates original CAR-T cells targeting several tumor-associated antigens (EGFR1, Her2, Her3 and Her-4), increasing the efficacy of CAR-T cell therapy; demonstrates novel CAR-CD4+CD25− and CD4+CD25+ T reg cells and highlights their functional differences; and shows novel CD47-CAR-T cells and regional delivery of CAR-T cells in solid tumors. The original view on quality of life analyses in clinical trial design proposed by Dr. Grace Dy et al. group could increase the safety and the outcome of Phase I clinical trials. Future novel CAR-T cells findings will be discussed in the next CAR-T issue in 2018.
